# CellTypeHGCN: a heterogeneous graph convolutional network for cell typing on single-cell RNA-seq data with reference TRN

**DOI:** 10.1093/bib/bbaf631.033

**Published:** 2025-12-12

**Authors:** Yongyu Long, Wenhao Zhang, Lan Cao, Xiaobing Huang, Ying Wang

## Abstract

Accurate cell annotation in single-cell RNA sequencing (scRNA-seq) data is a fundamental task for characterizing cellular states and identities. Existing approaches largely rely on gene expression similarity, but fail to capture the complex molecular dependencies that drive transcriptional programs. To address this, we propose CellTypeHGCN, a heterogeneous graph convolutional network (HGCN) framework thats integrates gene expression with transcriptional regulatory network (TRN) to explicitly model the regulatory architecture. TRN is naturally represented as heterogeneous graphs consisting of two regulatory element types, transcription factors and target genes, which are linked by directed regulatory relationships. Through HGCN, CellTypeHGCN can capture cellular representations while incorporating regulatory dependencies, thereby encoding the regulatory information that fundamentally determines cellular identity. Evaluations on six benchmark datasets demonstrate that CellTypeHGCN consistently outperforms seven state-of-the-art methods in cell type annotation. By integrating the prior TRN as the additional constraint, CellTypeHGCN can also robustly perform cross-platform cell type annotation and novel cell type identification. Moreover, the framework of CellTypeHGCN can be flexibly extended to single-cell ATAC-seq data, where it achieves superior performance to existing annotation tools. Overall, CellTypeHGCN provides a scalable, transferable, and mechanistically informed framework for accurate cell type annotation across different single-cell data modalities.

**References:**

1. Aibar S, et al. ‘SCENIC: single-cell regulatory network inference and clustering.’ Nature Methods 2017; **14**(11):1083–1086.

2. Yin Q, et al. ‘scGraph: a graph neural network-based approach to automatically identify cell types.’ Bioinformatics 2022; **38**(11):2996–3003.

3. Cha J and Lee I. ‘Single-cell network biology for resolving cellular heterogeneity in human diseases.’ Experimental & molecular medicine 2020; **52**(11):1798–1808.

